# The effect of Palm Oil Mill Effluent Final Discharge on the Characteristics of *Pennisetum purpureum*

**DOI:** 10.1038/s41598-020-62815-0

**Published:** 2020-04-20

**Authors:** Nurul Atiqah Osman, Farhana Aziz Ujang, Ahmad Muhaimin Roslan, Mohamad Faizal Ibrahim, Mohd Ali Hassan

**Affiliations:** 1 Department of Bioprocess Technology, Faculty of Biotechnology and Biomolecular Sciences, Universiti Putra Malaysia, 43400 UPM Serdang, Selangor, Malaysia; 2 Laboratory of Biopolymer and Derivatives, Institute of Tropical Forestry and Forest Products (INTROP), Universiti Putra Malaysia, 43400 UPM Serdang, Selangor, Malaysia

**Keywords:** Environmental biotechnology, Pollution remediation

## Abstract

Phytoremediation is one of the environmental-friendly and cost-effective systems for the treatment of wastewater, including industrial wastewater such as palm oil mill effluent final discharge (POME FD). However, the effects of the wastewater on the phytoremediator plants, in term of growth performance, lignocellulosic composition, and the presence of nutrients and heavy metals in the plants are not yet well studied. In the present work, we demonstrated that POME FD increased the growth of *P. purpureum*. The height increment of *P. purpureum* supplied with POME FD (treatment) was 61.72% as compared to those supplied with rain water (control) which was 14.42%. For lignocellulosic composition, the cellulose percentages were 38.77 ± 0.29% (treatment) and 34.16 ± 1.01% (control), and the difference was significant. These results indicated that POME FD could be a source of plant nutrients, which *P. purpureum* can absorb for growth. It was also found that the heavy metals (Al, As, Cd, Co, Cr, Ni and Pb) inside the plant were below the standard limit of the World Health Organization (WHO). Since POME FD was shown to have no adverse effects on *P. purpureum*, further research regarding the potential application of *P. purpureum* following phytoremediation of POME FD such as biofuel production is warranted to evaluate its potential use to fit into the *waste-to-wealth* agenda.

## Introduction

Pollution of soil and water caused by wastewater is one of the major global threats that our environment is facing today. One of the wastewater source is palm oil mill effluent final discharge (POME FD), which is a by-product of palm oil extraction process. Since Malaysia is the world’s second largest producer and exporter of palm oil, thus, a large amount of POME is generate annually^1^. It is estimated that for every tonne of crude palm oil produced, about 2.5 to 3.5 tonne of POME is generated^2^.

POME is considered as the main source of water pollution in Malaysia due to the high biochemical oxygen demand (BOD) and chemical oxygen demand (COD) that causes a reduction of the biodiversity and ability of aquatic ecosystem^3,4^. Furthermore, the damages to the river cannot be undone easily. Since POME is generated in huge amounts at a time, it is very difficult to manage, and the treatment of this wastewater is expensive. Consequently, the cheapest and easiest way for this wastewater disposal that have been practiced in Malaysia is by discharging the treated POME to the nearby river or stream^2,5,6^. However, Madaki and Seng^7^ noted that even treated POME (POME FD) still poses adverse effects on the environment; an observation also supported by the findings of Ibrahim *et al*.^8^. This is due to it still containing significant amount of organic matter. For that reasons, Ujang *et al*.^9^ has decided to further treat the POME FD using Napier grass constructed wetland in a process called phytoremediation, and thus, achieved 71.57% of COD and 83.59% of total suspended solid (TSS) reduction.

Phytoremediation is a process where plant naturally degrade, remove, or immobilize the contaminants in the wastewater^10^. This remediation approach is more cost effective, no production of by-product, low energy consumption and environmentally friendly compared with other method (*e.g*, adsorbent, reactors, and catalysis) to treat a large volume of wastewater^11^. One of the most common plant to remediate the industrial and agricultural wastewater is *P. purpureum*. For instance, Klomjek^12^ used two different varieties of *P. purpureum* for the treatment of swine wastewater. It was found that both varieties removed more than 70% of BOD and total Kjeldahl nitrogen (TKN). This method is feasible as *P. purpureum* has suitable features in treating wastewater. While it can grow fast and have high capability to absorb waters and nutrients from the soil to support the growth of the plant^12,13^, it can also grow on low nutrient and water supply^14,15^.

Even though *P. purpureum* is commonly used in phytoremediation of wastewater (including POME FD), no such study regarding the effects of the POME FD on the plant’s properties can be found. As POME FD contains significant amounts of nutrients (*e.g*., calcium, copper, magnesium, iron) and heavy metals (*e.g*., aluminium, arsenic, nickel), it could affect the application potential of the *P. purpureum* post-harvest, such as animal feed and biofuel production^16^. Therefore, in this study, *P. purpureum* was used as a phytoremediator to remediate POME FD and the growth performance of this grass grown on POME FD was evaluated. Then, the lignocellulosic composition, as well as nutrients and heavy metals concentration present in each part of plant after treatment with POME FD was determined and compared against control.

## Materials and Methods

### Palm oil mill effluent final discharge analysis

The POME FD was collected from Felda Pasoh Palm Oil Mill, Negeri Sembilan, Malaysia. The collected sample was stored in airtight plastic bottles at 4 °C prior using. The BOD, COD, pH, colour, TSS, and ammonia concentration were analysed using the American Public Health Association (APHA) Standard Methods for the Examination of Water and Wastewater^17^. The total Kjeldhal nitrogen (TKN) was analysed using the Simplified TKN method. The nutrients and heavy metals concentration were determined by inductive coupled plasma-optical emission spectrometry (ICP-OES) and inductive coupled plasma-mass spectrometry (ICP-MS).

### Experimental design

**Figure 1:**
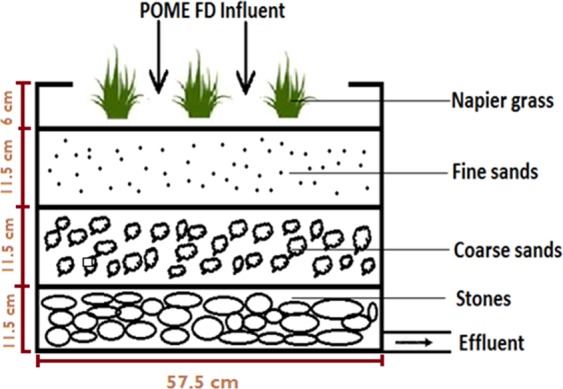
Design of Napier grass (*Pennisetum purpureum*) wetland system (Adapted from Ujang *et al*.^9^).

The sample of *P. purpureum* (common cultivar) was obtained from the Biomass Technology Laboratory (BTL), Universiti Putra Malaysia. The wetland system was constructed as shown in Fig. 1. Each system was constructed from a 60 L (57.5 cm × 40.5 cm × 38.5 cm) plastic container. The containers were filled with fine sands, coarse sand, and crushed run stones at a ratio of 1:1:1^9^. The sand, coarse sand and crushed run stones were sun dried for 1 week to reduce the moisture content to almost 0%. No microorganism was added into the media prior to the experimental procedure. All media used was kept dried and show zero microorganism activity during preparation. The two months old *P. purpureum* was then transferred into the wetland system. Each treatment and control consisted of three replicates of the wetland system, where each system was planted with four grasses. The treatment systems were supplied with 5 L of POME FD per day, whereas the control was supplied with rain water at 5 L every day. Rain water was used as a control to mimic the natural condition of wild *P. purpureum* since in the wild, this robust plant only depends on rain water to grow.

### Plant sampling, harvesting and sample preparation

The plant were monitored for 8 weeks; during which the growth parameter (height, number of leaves and number of tillers) were evaluated weekly. The plant height was measured using a flexible measuring tape, starting from ground-height to the tip of the longest leaves. Number of leaves and tillers per plant was made by a visual count of the green leaves. The plant were then harvested at the end of week 8. The components of the plant were separated, including stems and leaves. The stems were pressed using a sugar cane pressing machine to yield the juice, while for the leaves, they were chopped to 6–8 cm size. The samples were then sun-dried for three days until moisture content was less than 10% and subsequently ground by a hammer mill with 2 mm sieve. Then, the grounded samples were kept dry and stored in airtight plastic bags. The juice was kept at −4 °C.

### Lignocellulosic composition

Lignocellulosic biomass has three major components which are cellulose, hemicellulose, and lignin, along with moisture content, extractives and ash. The moisture content of the biomass was determined by using automated infrared moisture analyser with an aluminium pan (MX-50 MODEL, A&D COMPANY, JAPAN). The total extractives of the sample were determined through two-step extraction, as outlined by the National Renewable Energy Laboratory method, NREL/TP-510-42619^18^. The determination of the lignocellulosic composition of *P. purpureum* was performed using acid hydrolysis method and High Performance Liquid Chromatography (HPLC) following the National Renewable Energy Laboratory method, NREL/TP-510-42623^19^. The determination of ash in the biomass sample was carried out following the National Renewable Energy Laboratory method, NREL/TP-510-42622^20^.

### Elemental analysis

The digestion of dried stems and leaves was performed following the improved HNO_3_/H_2_O_2_ digestion method described by Pequerul *et al*.^21^ with slight modification. Briefly, 5 mL of HNO_3_ was poured into a 250 mL dry flask containing 0.5 g of sample, then stirred until all samples were completely wet. Next, 4 mL of 33% H_2_O_2_ was added and gently stirred in a well-ventilated hood. This flask containing sample was then heated in a boiling water bath (100 °C) until a strong effervescence was produced. When brown fumes were less dense (10–12 min), the solution was allowed to cool. A slightly yellow dissolution and small quantity of white solid in suspension would remain. The solution was then filtered and washed with 5 mL of (1:1 v/v) HCl (density 1.18 g/mL). Next, the solution was diluted with distilled water up to 25 mL until a clear solution was formed. The elements’ (micronutrients, macronutrients, heavy metals) concentrations in the stems and leaves digested solution as well as in the juice were determined using ICP-MS.

## Results and Discussion

### Characteristics of palm oil mill effluent final discharge

The physico-chemical properties of POME FD (treatment) and rain water (control) are shown in Table 1. POME FD is characterized as slightly basic with a pH value of 8.72 ± 0.35. It can be noted that the BOD, COD and TSS of the POME FD is higher compared to rain water. This is associated with the presence of higher biodegradable organic waste in the wastewater. Additionally, the colour and TKN recorded in POME FD was also higher than the rain water.

**Table 1:** Physico-chemical properties of POME FD (treatment) and rain water (control).

Parameter	Unit	POME FD	Rain water
BOD	mg/L	36.50 ± 6.24^a^	1.62 ± 0.01^b^
COD	mg/L	987.17 ± 4.01^a^	3.70 ± 0.01^b^
pH	—	8.72 ± 0.35^a^	7.59 ± 0.32^b^
TSS	mg/L	37.63 ± 3.71	n.d.
Colour	PtCo	590.00 ± 9.48^a^	1.30 ± 0.92^b^
Total Kjeldhal nitrogen (TKN)	mg/L	153.25 ± 0.50^a^	2.09 ± 0.02^b^

^a,b^Mean significant differences at *p*-value < 0.0001, according to Duncan’s Multiple Range Test. *n.d. = not detected.

The mean ± SD values of various nutrients and heavy metals of POME FD and the control are presented in Tables 2 and 3. Out of the nutrients, Mg, Ca, and K have the highest concentration in POME FD were 320,639.44 ± 3,379.20 ppb, 75,721.30 ± 11,302.60 ppb and 9,571.31 ± 1,537.03 ppb, respectively. This may be due to the fact that these nutrients are regarded as essential supplements for enhancing plant growth and productivity of the oil palms, and thus, can be beneficial for the *P. purpureum* as well^22^.

**Table 2:** Micronutrient and macronutrient concentrations in POME FD (treatment) and rain water (control).

Element	POME FD (ppb)	Rain water (ppb)
**Macronutrient**		
Calcium (Ca)	75,721.30 ± 11,302.60^a^	8,210.92 ± 284.56^b^
Potassium (K)	9,571.31 ± 1,537.03^a^	3,816.69 ± 93.71^b^
Magnesium (Mg)	320,639.44 ± 3,379.20^a^	980.28 ± 32.85^b^
**Micronutrient**		
Copper (Cu)	6.69 ± 0.32^a^	0.38 ± 0.03^b^
Iron (Fe)	514.76 ± 9.91^a^	60.48 ± 1.75^b^
Manganese (Mn)	10.86 ± 0.70^b^	14.29 ± 0.78^a^
Zinc (Zn)	3.64 ± 0.17^a^	1.34 ± 0.48^b^

^a,b^Mean significant differences at *p*-value < 0.0001, according to Duncan’s Multiple Range Test.

**Table 3:** Heavy metals concentrations in POME FD (treatment) and rain water (control).

Element	POME FD (ppb)	Rain water (ppb)
Aluminium (Al)	100.33 ± 8.91^a^	30.65 ± 2.26^b^
Arsenic (As)	35.73 ± 0.04^a^	0.89 ± 0.01^b^
Cadmium (Cd)	0.053 ± 0.02^b^	0.074 ± 0.05^a^
Cobalt (Co)	2.20 ± 0.08^a^	0.14 ± 0.11^b^
Chromium (Cr)	8.27 ± 0.13^a^	2.89 ± 0.24^b^
Nickel (Ni)	11.65 ± 0.48^a^	1.17 ± 0.17^b^
Lead (Pb)	0.19 ± 0.01^a^	0.08 ± 0.01^b^

^a,b^mean significant differences at *p*-value < 0.0001, according to Duncan’s Multiple Range Test.

Besides nutrients, the presence of heavy metals in POME FD could probably be due to several factors. For example, it can come from trace metal contents of the crops since heavy metals can be bioaccumulated by the plant from contaminated environment^23,24^, contamination during the digestion process, or leaching from the processing equipment over time^25^. Based on Table 3, the presence of Al in POME FD was the highest (47.48% higher than arsenic, which is the second highest) as compared to other metals. This could be explained by the fact that, in palm oil mills, coagulants were used in the boiler water treatment to reduce the turbidity, organic substances, and colour of raw water. Most common coagulants are aluminium-based, such as aluminium sulphate (alum), sodium aluminate, and polyaluminium chloride (PAC)^26^. Therefore, the water treated with these coagulants would eventually be contaminated with aluminium and then discharged together with other wastewater from the mill as POME FD.

Despite the presence of heavy metals in POME FD, their concentrations were relatively low (except Al) and fulfilled the conditions for the discharge of industrial effluent of standards A and B, from the Environmental Quality (Industrial Effluents) Regulations 2009 that have been prescribed by the Department of Environmental (DOE), Malaysia^27^. However, the resulting bioaccumulation of heavy metals in plants supplied with this wastewater makes the determination of heavy metal contents as an important parameter to be monitored. This is to avoid toxicity to the phytoremediator plants during treatment period as well as for safe consumption by animal post-harvest.

### *P. purpureum* physical properties evaluation

**Figure 2:**
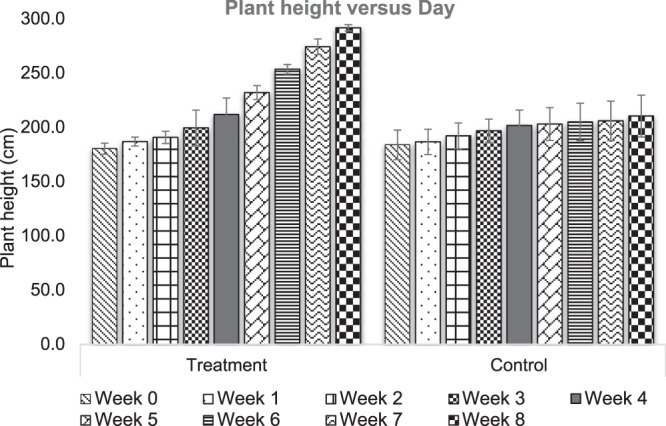
The height of common cultivar of *P. purpureum* grown in POME FD (treatment) and rain water (control) for 8 weeks.

**Figure 3:**
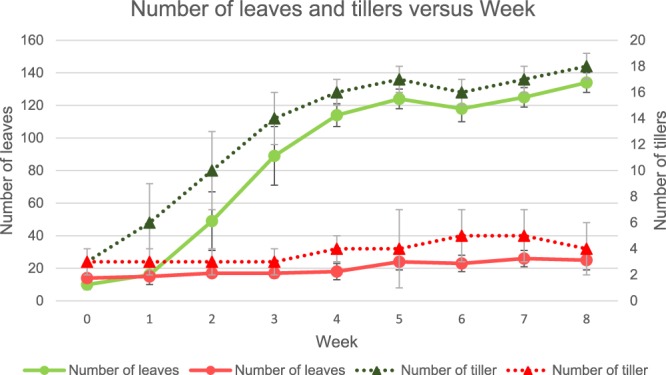
The number of leaves and tillers of common cultivar of *P. purpureum* grown in POME FD (treatment) and rain water (control) for 8 weeks.

The growth parameters, which included the height, the number of leaves, and number of tillers of *P. purpureum*, were evaluated every week for a duration of 8 weeks. Generally, it was clear that there was an increasing trend in both treatment and control from week 0 to week 8 as can be seen in Fig. 2. From the statistical analysis, the height of the treatment plant was significantly higher than control with *p*-value < 0.0001. From week 0 to week 8, the percentage increment in height for treatment was 61.72 ± 2.36% while for control was only 14.42 ± 2.11%. In addition, based on Fig. 3, it can be seen that there is an increasing trend in the leaves and tillers’ number for both control and treatment plant. The treatment plants yielded better growth with range of 3 to 5 times higher number of leaves and tillers compared with control.

**Figure 4:**
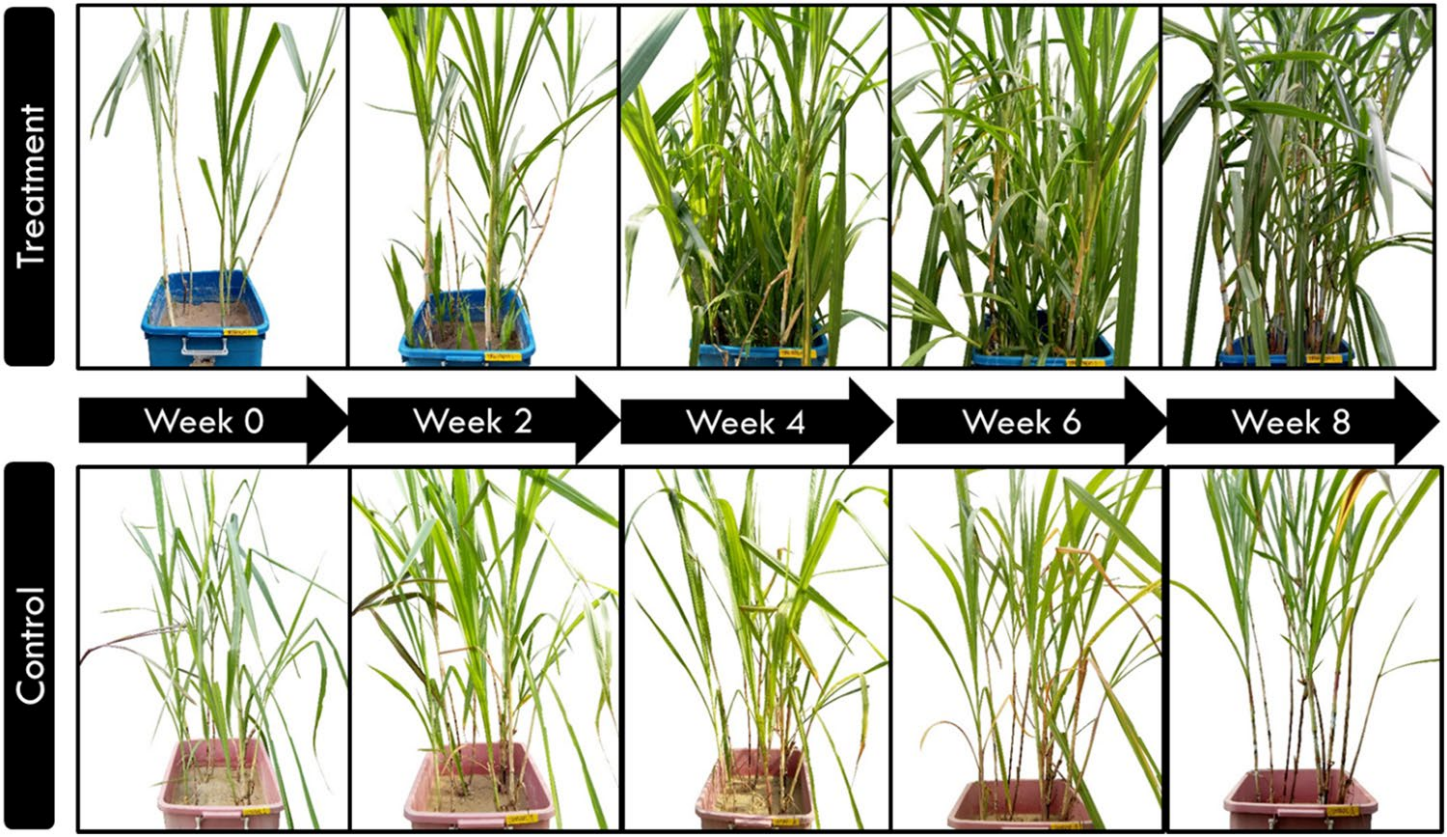
Control and treatment systems of common cultivar of *P. purpureum* from week 0 to week 8.

The growth of a plant is very reliant on the uptake of mineral nutrient^28^. For example, iron (Fe) that found to be approximately 514.76 ± 9.91 ppb in POME FD, is involved in chlorophyll synthesis and essential for the maintenance of chloroplast structure and function^29^. On the other hand, potassium (K) (9571.31 ± 1537.03 ppb in POME FD) functions as the major osmoticum of the cells, controlling cell expansion, plasma membrane potential and transport, pH value, and many other catalytic processes^28^. The deficiency of these nutrients will lead to a reduction in growth and an increase in pathogen susceptibility. Since the concentration of these nutrients in POME FD is higher than rain water (Table 2), this suggest that these nutrients may play a part in encouraging a better growth for the treatment plants. Figure 4 further illustrates the comparison in plant growth between treatment and control system of *P. purpureum*.

Besides that, nitrogen may provide the biggest influence on the number of leaves and tillers in this experiment. An adequate supply of nitrogen to plants will allow the plants to produce more tillers which lead to more shoots and leaves^30^. Plants that receive a high supply of nitrogen, for example 153.25 mg/L TKN in POME FD (Table 1), will produce higher mesophyll cells’ number, relative to epidermal cells^31,32^. This condition could produce a thicker structure of leaves, given the importance of mesophyll cells for photosynthesis. Therefore, the rate of photosynthesis is higher in plant grown under high nitrogen content. The higher photosynthesis rate will then result in an increase in relative growth rate^33^. These support the observation of high percentage increment in the height of treatment plants (61.72 ± 2.36%) as compared to that of control (14.42 ± 2.11%) observed in this study.

Therefore, it can be concluded that POME FD could supply nutrients that are essential to encourage plant growth. This is also supported by the findings of Mohammad and Ayadi^34^ which stated that wastewater irrigation increased the yield of corn and vetch.

### Lignocellulosic characterization of *P. purpureum* grown on palm oil mill effluent final discharge

The lignocellulosic composition distribution in treatment and control systems are shown in Table 4. The results of lignocellulosic composition obtained in the present work are considered reliable since they are within the range of general grass biomass as described by Sun and Cheng^35^, and Howard *et al*.^36^, where the percentage range are 25–40%, 25–50% and 10–30% for cellulose, hemicellulose, and lignin respectively.

**Table 4:** Summary of lignocellulosic constituent of *P. purpureum* grown on POME final discharge (treatment) and rain water (control).

		Total extractives (%)	Ash (%)	Total lignin (%)	Cellulose (%)	Hemicellulose (%)
Treatment	Stem	16.25 ± 0.86	1.17 ± 0.18	12.43 ± 0.64	38.77 ± 0.29	31.38 ± 0.35
	Leaves	30.12 ± 1.02	4.36 ± 0.54	11.65 ± 0.92	25.67 ± 1.12	28.29 ± 0.53
Control	Stem	15.49 ± 1.13	2.46 ± 0.25	11.14 ± 0.79	34.16 ± 1.01	36.75 ± 0.22
	Leaves	29.44 ± 1.29	4.21 ± 0.18	9.73 ± 2.32	27.67 ± 0.28	30.05 ± 1.04

From Table 4, cellulose content in stem for treatment system (38.77 ± 0.29%) was higher than control (34.16 ± 1.01%). There are various factors that contribute to cellulose content in plant, for instance: water availability, heat, light, and nutrient availability^37^. In the present work, the plant in both systems had the same external condition except for the nutrient availability due to the different types of water feed (POME FD for treatment system and rain water for control system). One of the nutrients that affect the cellulose content in plants is potassium (K)^38^. K is essential for the performance of multiple enzymatic functions as well as on carbohydrate metabolism and photosynthesis^39,40^. When plants obtain sufficient amount of K, the synthesis of large biomolecules such as cellulose are markedly increased^38^. This is because, K enhances enzyme actions that aids in photosynthesis and food formation. As the photosynthesis rate of plant increases, the cellulose deposition in plants also increases. Since POME FD had a higher content of K than rain water (refer Table 2), the plants in the treatment system consequently obtained more than sufficient amount of K, thus having a higher cellulose content than in control plants.

In contrast to cellulose, the hemicellulose content in treatment plants (31.28 ± 0.35%) was lower as compared to control (36.75 ± 0.22%). Several studies found that the concentration of hemicellulose in plants decreased when fertilizers are used to enhance plant growth^41–43^. This is because, high soil nutrient availability will not only increase plant growth, but also carbon sink (C-sink) which reduces carbon reserves^43^. This explains why the concentration of hemicellulose was low since hemicelluloses play a role as carbon reserves for plants^44^.

Lignin is the third major element of lignocellulosic biomass. An increase in lignin content often leads to an increase in biomass rigidity^45^. However, the results obtained from the present work revealed that there was no significant difference between the treatment and control systems.Taken together, it can be concluded that the nutrients contained in POME FD did affect the structural carbohydrate composition of *P. purpureum*, as evidenced between the treatment and control systems. This is important because lignocellulosic composition will affect biosugars production from the biomass of the harvested *P. purpureum*.

### Elemental characterization of micronutrients, macronutrients and heavy metals in *P. purpureum* grown on palm oil mill effluent final discharge

**Figure 5:**
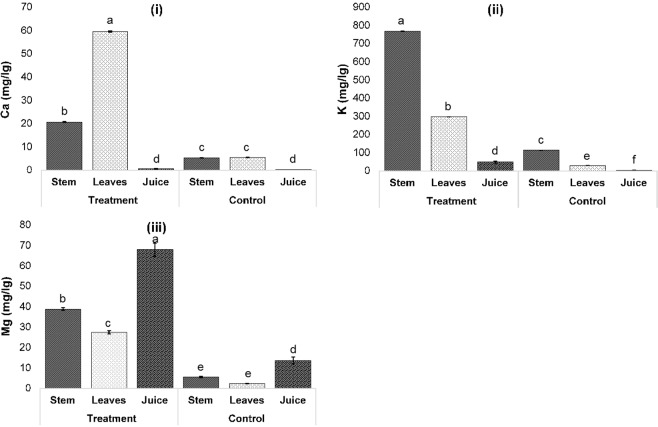
Micronutrients, Ca (**i**), K (**ii**) and Mg (**iii**) concentrations (mg/kg) in stem, leaves and juice of *P. purpureum* grown on POME FD (treatment) and rain water (control). ^a,b,c,d,e,f^Mean significant differences between treatment and control at a different part of plant, at *p*-value < 0.0001, according to Tukey’s Least Squares Means Test.

**Figure 6:**
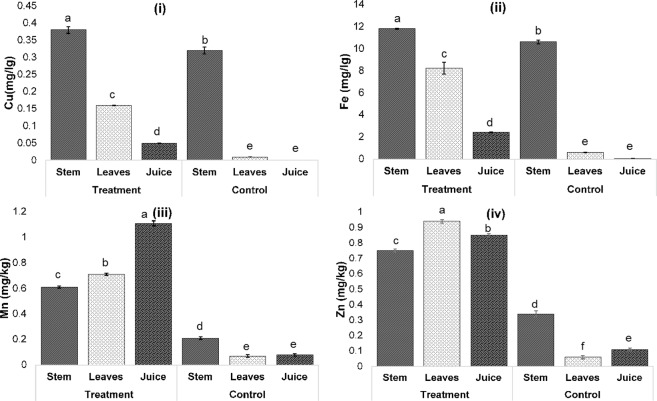
Macronutrients, Cu (**i**), Fe (**ii**), Mn (**iii**) and Zn (**iv**) concentrations (mg/kg) in stem, leaves and juice of *P. purpureum* grown on POME FD (treatment) and rain water (control). ^a,b,c,d,e,f^Mean significant differences between treatment and control at a different part of plant, at *p*-value < 0.0001, according to Tukey’s Least Squares Means Test.

The micronutrients and macronutrients’ concentrations in the parts of *P. purpureum* for treatment and control systems are presented in Figs. 5 and 6. As expected, it can be seen that all micronutrients and macronutrients in *P. purpureum* grown on POME FD were higher than in control. These outcomes agree with the findings of Ali *et al*.^46^ which revealed that nutrient contents (N. P. and K) in the *Melia azedarach* were higher when grown on wastewater as compared to tap water.

From Fig. 5, the order of Ca concentration in each part of the plant was leaves > stems > juice, for both treatment and control systems. Root system acquired Ca from the soil solution, which is then translocated to the shoot via xylem^47^. This could be the reason why the concentration of Ca in the leaves was higher as compared to in stems and juice. Moreover, the leaves can absorb much more Ca from a high-Ca environment as compared to from normal environments^48^. This statement supports the finding obtained in the present work where the Ca content in the treatment system was higher than the control system because of the Ca availability from the POME FD (refer Table 2). The function of Ca is to hold the plant cell walls by forming cross-link with pectin residues^47^. Therefore, Ca deficiency in the plant will lead to distorted growth of new tissues, for instance the young leaves and shoot tips, due to the disrupted cell wall formation^47^. As illustrated in Fig. 3, the treatment system yielded a higher number of leaves, and this was further reflected in the higher content of Ca in the leaves of *P. purpureum* in the treatment system.

Besides Ca, K and Fe are also important elements for plant growth. However, in contrast with Ca, the order of K and Fe concentrations in each part of the plants for both system was stems > leaves > juice **(**Figs. 5 and 6**)**. Similar results were obtained by Halder *et al*.^49^ who stated that the accumulation of K among the vegetable parts was higher in the stems (ranged from1.64 ± 0.04% to 9.96 ± 0.04%) as compared to in the leaves (ranged from 0.14 ± 0.02% to 4.39 ± 0.01%). However, the distribution of Fe in the plant obtained in the present work contradicted with Olowu *et al*.^50^ who stated that leaves had a higher Fe content as compared to stems. This could be due to the high level of several minerals accompanying Fe that may involve in the interaction with Fe in the soil, thus lowering the mobility of Fe as well as inhibiting the absorption of Fe by the plant^51,52^. As stated by Liu *et al*.^53^, Co in the system decreased Fe concentration in leaves by about 80%.

On the contrary, the concentration of Mg showed a decreasing trend in the order of juice > stems > leaves **(**Fig. 5**)**. This could be due to the solubility of Mg. Mg is essential for photosynthesis since it is a constituent of every chlorophyll molecule^54^. In the plant, 80% of the total Mg are present in mobile forms while the other 20% are bound to chlorophylls^55,56^. Because of the soluble and mobile characteristics of Mg, it can be easily extracted from the stems along with the juice via pressing.

From Table 5, it is apparent that the heavy metals’ concentrations in treatment were higher than in control. This trend was also observed in the concentrations of micronutrients and macronutrients in the plants as previously discussed. This outcome is supported by García *et al*.^57^ who demonstrated that the concentrations of heavy metals in the plants correlated to the concentrations of heavy metals in the water. Therefore, high concentrations of heavy metals in wastewater will cause correspondingly high concentrations of heavy metals in the plant.

**Table 5:** Heavy metal concentrations (mg/kg) in parts of common cultivar of *P. purpureum* grown on palm oil mill effluent final discharge (treatment) and rain water (control), and comparison with the standards.

Element	This study						,Range of heavy metal in plants^62,63^	Indian standards in food^61^	Permissible value in plant^60^
	Treatment			Control					
	Stem	Leaves	Juice	Stem	Leaves	Juice			
Al	2.10 ± 0.07^b^	4.36 ± 0.07^a^	0.21 ± 0.01^d^	1.45 ± 0.04^c^	0.21 ± 0.01^d^	0.02 ± 0.01e-1^e^	—	—	—
As	0.37 ± 0.02^a^	0.16 ± 0.01^b^	0.01 ± 0.05e-2^d^	0.05 ± 0.02e-1^c^	0.02 ± 0.01e-1^d^	n.d.	0.02–7.00	1.10	—
Cd	n.d.	n.d.	n.d.	n.d.	n.d.	n.d.	0.10–2.40	1.50	0.02
Co	0.01 ± 0.02e-2	n.d.	n.d.	n.d.	n.d.	n.d.	0.05–0.50	—	—
Cr	n.d.	n.d.	n.d.	n.d.	n.d.	n.d.	0.20–1.00	20.00	1.30
Ni	0.03 ± 0.06e-2^c^	0.04 ± 0.06e-2^b^	0.02 ± 0.04e-3^d^	0.06 ± 0.04e-2^a^	n.d.	n.d.	1.00	1.00	10.00
Pb	0.04 ± 0.08e-2^a^	0.03 ± 0.05e-2^b^	n.d.	0.01 ± 0.02e-2^c^	n.d.	n.d.	1.00–13.00	2.50	2.00

^a,b,c,d,e,f^Mean significant differences between treatment and control in different parts of plant at *p*-value < 0.0001 according to Tukey’s Least Squares Means Test.

The concentrations of heavy metals, especially Al, were higher in POME FD, which raises concerns on the accumulation of these metals in the plants. As shown in Table 5, the Al content was in the range of 0.21–4.36 mg/kg in treatment while only 0.02–1.45 mg/kg in control. On the other hand, the other heavy metals (As, Cd, Co, Cr, Ni and Pb) were relatively in low concentrations because of the initial low concentrations of these metals in the POME FD and rain water (Table 3). The high Al content could potentially be a major concern to the plant growth because it can cause toxicity to the plants. The main consequence of Al exposure is the inhibition of root growth^58^. The inhibition of root growth may consequently lead to the reduction in plant growth since roots are essential for water and nutrient absorption by the plants. However, as can be seen in Fig. 3, the plant grown on POME FD was healthy and grew steadily. In addition, Al will become toxic to plant when their concentration is higher than 2–3 ppm with soil pH below 5.5^59^. This explains why the growth of *P. purpureum* grown on POME FD was unaffected since POME FD is a basic wastewater with pH value of 8.72 and the Al concentration in POME FD is only 0.1 ppm.

The results of heavy metal concentrations inside the plant were compared with WHO standards^60^ and Indian standards of heavy metals in foods^61^ **(**Table 5**)**. All heavy metals were below the standard limits with the values were in the range of commonly found heavy metal concentrations in plants as stated by Misra and Mani^62^, and Nagajyoti *et al*.^63^. Although these concentrations were within the acceptable and standard levels, the plants used for the phytoremediation of POME FD still need to be monitored since prolonged exposure to these heavy metals might lead to their accumulation inside the plants, which increases their concentration to a toxic level that would negatively affect the plant growth.

## Conclusions

In conclusion, the nutrient-rich POME FD did not have adverse effects on the growth of *P. purpureum*. Instead, the POME FD was able to supply the plant with the essential nutrients that promoted its growth, such as Fe, K and N. It was also found that the heavy metals inside the plant were below the standard limit prescribed by the WHO. Interestingly, POME FD also increased the cellulose content in the treatment system. This opens up an array of potential applications of *P. purpureum* post-phytoremediation, such as feedstock for the production of biofuels. Therefore, instead of disposing the plants post-phytoremediation, utilizing this waste in a more profitable means will not only make wastewater treatment more environmentally friendly, but also more cost effective. Further research regarding the application of *P. purpureum* post-phytoremediation of POME FD is hence warranted to evaluate its potential use to fit into the *waste-to-wealth* agenda.
